# Mind the Gap: Mitochondria and the Endoplasmic Reticulum in Neurodegenerative Diseases

**DOI:** 10.3390/biomedicines9020227

**Published:** 2021-02-23

**Authors:** Nuno Santos Leal, Luís Miguel Martins

**Affiliations:** MRC Toxicology Unit, University of Cambridge, Cambridge CB2 1QR, UK

**Keywords:** mitochondria–ER contact sites (MERCS), mitochondria–ER associated membrane (MAM), neurodegeneration, neurodegenerative diseases, Alzheimer’s disease, Parkinson’s disease, amyotrophic lateral sclerosis, frontotemporal dementia

## Abstract

The way organelles are viewed by cell biologists is quickly changing. For many years, these cellular entities were thought to be unique and singular structures that performed specific roles. However, in recent decades, researchers have discovered that organelles are dynamic and form physical contacts. In addition, organelle interactions modulate several vital biological functions, and the dysregulation of these contacts is involved in cell dysfunction and different pathologies, including neurodegenerative diseases. Mitochondria–ER contact sites (MERCS) are among the most extensively studied and understood juxtapositioned interorganelle structures. In this review, we summarise the major biological and ultrastructural dysfunctions of MERCS in neurodegeneration, with a particular focus on Alzheimer’s disease as well as Parkinson’s disease, amyotrophic lateral sclerosis and frontotemporal dementia. We also propose an updated version of the MERCS hypothesis in Alzheimer’s disease based on new findings. Finally, we discuss the possibility of MERCS being used as possible drug targets to halt cell death and neurodegeneration.

## 1. The Beginning: Cells, Organelles and Organelle Contact Sites

The Earth is 4.5 billion years old, and life on our planet began approximately 3.8 billion years ago. Since there is no fossil record of the beginning of life on our planet, we can only speculate about the origin of the first macromolecules and lifeforms. Evolutionary biologists believe that the first cells originated after a phospholipid membrane encapsulated a self-replicating RNA [1,2]. With time, this primordial cell evolved, becoming more complex, and evolving into the ancestors we know as organelles and prokaryotic cells. These first cells were anaerobic and obtained energy through the breakdown of organic molecules in the absence of oxygen [2]. Therefore, natural selection favoured cells that produced the most energy and replicated the fastest. However, bacterial growth is limited by its geometry. Every time a bacterium grows, its volume/surface area ratio decreases (with the exact value depending on bacterial shape), resulting in a decrease in its respiratory efficiency due to an increase in energy demand (volume) relative to energy production (surface area). Changing shape and folding membranes to create sheets and villi enabled cells to overcome this reduced respiratory capacity. However, these complex processes made them extremely fragile with difficulty replicating accurately, and thus, these cells were not selected [3]. Pre-eukaryotic cells originated between approximately one and two billion years after the first cells emerged upon the engulfment of a facultative anaerobe, α-proteobacterium, by an archaebacterium, developing an endosymbiotic relationship and allowing them to evolve together [2,4]. This symbiosis provided several advantages over other cells. First, with an energy producer inside the cell, these new eukaryotic cells could lose their highly impermeable cell wall without losing the chemical gradient necessary for energy production. This allowed their outer membrane to specialize, with functions such as cell signalling, phagocytosis and movement. These pre-eukaryotic bacteria were also able to grow larger since they could increase their energy efficiency by merely increasing the number of α-proteobacterium energy producers without affecting the total volume of the cell. In fact, eukaryotic cells are on average 10,000- to 100,000-fold larger than bacteria [3]. Around this time, the levels of oxygen on Earth started to increase. The engulfed α-proteobacterium could convert oxygen into energy, which allowed a sixfold increase in energy production upon glucose degradation in the new pre-eukaryotic cell, which conferred a competitive advantage over other organisms [5]. Eventually, this endosymbiotic relationship led to the precursor of eukaryotic cells as we know them today, with α-proteobacteria being the precursors of mitochondria. Similar processes occurred with chloroplasts, which are cellular organelles originating from photosynthetic bacteria [1]. On the other hand, the nucleus and endoplasmic reticulum (ER) might have originated from plasma-membrane invaginations [4]. In addition to nuclei, mitochondria are the only other organelles in animal cells in which DNA can be found—mitochondrial DNA (mtDNA). However, most of the DNA (approximately 95 to 99.5%) from primordial mitochondria was transferred to the nuclear genome. In fact, this transfer did not occur at a single time point, as indicated by the human genome project, which has shown that at least 354 independent DNA-transfer events occurred from mitochondria to nuclei. Interestingly, different species seem to have maintained the same genes inside of mitochondria, despite their different evolutionary paths, suggesting that this transfer does not occur randomly. Several theories have been proposed to explain why the whole mitochondrial genome has not been integrated into the nuclear genome, including the fact that the former codes for proteins that are either large or too hydrophobic, or the fact that mtDNA allows a response to changes in mitochondrial respiration that is faster than that of a retrograde response. Even today, after billions of years, mitochondria are somewhat independent. For example, mitochondria and the host cell divide independently, with mitochondria able to replicate inside of host cells [3]. Other organelles have also been shown to influence mitochondrial function and ultrastructure. For example, the ER has been shown to mark the place where mitochondrial division occurs (discussed below). Currently, eukaryotic cells are complex and contain several organelles. These organelles are believed to be individual entities delimited by well-defined membranes with unique features designed to allow for specific cellular functions. However, modern advances in technology, such as electron microscopy and subcellular fractionation, as well as the discovery of the secretory pathway and clathrin-coated vesicles, have led researchers to question whether organelles are truly single and independent cellular entities [6,7,8]. Currently, we know that organelles form highly complex networks, and their crosstalk is essential for their normal development and function, as well as for cell homeostasis.

Although a fairly new area, the field of organelle contact sites has been exploding, and several organelle contact sites have been identified, including plasma membrane–mitochondria, ER–Golgi, and mitochondria–peroxisomes [9,10]. Generally, contact sites are classically defined as membranes of different organelles in close apposition. However, due to momentum and increasing interest in the field, a more concrete definition of contact sites has recently been established. The following criteria used to define a contact site have been proposed: (1) there must be a tether between two bilayer or monolayer membranes, (2) there must be no fusion between the membranes, (3) there must be a specific function for this contact site and (4) there must be a defined proteome and/or lipidome [11]. These organelle contact sites are important for normal cell functioning, and alterations in these sites have been reported to be associated with several diseases, including cancer, obesity, diabetes and infection [12,13,14]. In this review, we explore the roles of one type of organelle contact site, mitochondria–ER contact sites (MERCS), in neurodegenerative disorders (NDs). In particular, we provide an updated overview of the relevant molecular composition and the dysregulated MERCS-related biological pathways in Alzheimer’s disease (AD), with a brief overview of these factors in Parkinson’s disease (PD) and amyotrophic lateral sclerosis (ALS)/frontotemporal dementia (FTD).

## 2. Mitochondria–ER Contact Sites and Mitochondria-Associated ER Membranes

MERCS were first observed in the 1950s by Bernhard and colleagues [15,16], while the first biochemical fraction enriched at this juxtaposition was obtained 15 years later [17,18,19,20]. However, it was not until the 1990s that Jean Vance identified the first functional role of connected ER and mitochondria, showing that phosphatidylserine (PSer), phosphatidylethanolamine (PE) and phosphatidylcholine (PC) were synthetized in a subcellular fraction enriched with mitochondria-associated ER membrane (MAM) [21,22]. The terms MERCS and MAM are often used interchangeably; however, this usage is in accurate. While MERCS refers to the ultrastructure and tethering architecture of the contact sites, MAM refers to the biochemical properties of these contacts, and sometimes, MAM is also used to refer to the specialized lipid raft-like domain in the ER that interacts with mitochondria and that is pulled down via subcellular fractionation [22,23,24]. Currently, over 100 proteins have been shown to either have a structural or functional/biochemical role in these contacts, including calcium (Ca^2+^) shuttling from the ER to mitochondria, autophagosome formation, reactive oxygen species (ROS) signalling and phospholipid metabolism. These contacts are thought to cover from approximately 5 to 12–20% of the mitochondrial surface, depending on the type of cells and their metabolic stage [23,24,25]. Here, we focus on the tethers and functional roles that have been connected with AD, PD and ALS/FTD. Therefore, a detailed overview of the ultrastructure of MERCS and their composition is beyond the scope of this review, but has been presented in different publications, including those by Prinz et al., Schon et al. and Pailluson et al. [13,26,27].

### 2.1. Mitochondria

Structurally, mitochondria are composed of two lipid bilayer membranes (the outer and inner mitochondrial membranes—the OMM and IMM, respectively) and two aqueous compartments (the intermembrane space (IMS) and matrix). Each structure has a specific composition and role in maintaining normal mitochondrial and cell functioning [1]. The OMM delimits a mitochondrion and is the basis of the organelle shape and morphology. The OMM contains a high number of integral proteins that allow the passage of molecules as large as 5000 daltons to and from mitochondria. Larger molecules need to be selectively transported by the translocase of the outer membrane (TOM). IMM is characterized by enrichment with cardiolipin, a phospholipid with four fatty-acid chains instead of the standard two fatty-acid chains, making this membrane extremely impermeable. This impermeability allows the formation of mitochondrial membrane potential (ΔΨ_m_) since it sequesters the protons released during oxidative phosphorylation (OXPHOS) into the IMS. Invaginations of the IMM into the matrix are called cristae, and they harbour complexes that form the electron-transport chain (ETC). Transport across this membrane is performed via the translocase of the inner membrane (TIM) or ion transporters and is dependent on the presence of a ΔΨ_m_ [1,28]. IMS is the aqueous compartment between the OMM and IMM, and it is known for storing protons released during OXPHOS and for regulating mitochondrial protein import [1,29]. In the mitochondrial matrix, numerous chemical reactions occur, including the tricarboxylic acid (TCA) cycle, reduction of nicotinamide adenine dinucleotide (NAD) to NADH and β-oxidation. It is also in the mitochondrial matrix where mtDNA is harboured and where mitochondrial transcription and translation occur. Even though mtDNA encodes some mitochondrial proteins, the majority of mitochondrial proteins are encoded by nuclear DNA in the cytosol or ER and need to be imported via a signalling peptide [1].

Mitochondria are best known for their role in the production of adenosine triphosphate (ATP), a biological energy molecule. ATP can be formed in different pathways, with glycolysis and OXPHOS being the main sources of ATP production during normal cell functioning. However, glycolysis produces a very small amount of ATP compared to OXPHOS. During glycolysis, glucose (six carbon molecules) is only partially degraded, with one covalent bond being broken, forming two molecules of pyruvate (three carbon molecules). Pyruvate, together with coenzyme A (CoA), forms acetyl-CoA and allows the continuation of its degradation inside mitochondria in the TCA cycle. During this process, large amounts of reduced NADH and flavin adenine dinucleotide (FADH_2_) are formed. NADH and FADH_2_ act as electron carriers, transporting electrons to the ETC where they will be transferred between different complexes (I to IV). During this electron transfer and reduction of complexes, protons are transferred to the IMS, where they accumulate and create the ΔΨ_m_ due to the difference in the electrochemical gradient between IMS and the matrix. Due to the difference in this gradient and the impermeability of the IMM, protons can only return to the matrix via complex V (F_O_F_1_-ATP synthase), creating kinetic energy that induces the rotation of this complex and phosphorylation of ADP into ATP [1].

### 2.2. The Endoplasmic Reticulum

The endoplasmic reticulum is one of the largest organelles, expanding throughout the whole cell from the nucleus to the plasma membrane. Similar to mitochondria, the ER is present in whole eukaryotic cells and is composed of a connected phospholipid bilayer membrane that is shaped like tubules or flattened sacs. This membrane separates the ER lumen, which is connected to the nucleus, and the cytosol. The major functions of the ER are lipid (smooth ER–SER) and protein (rough ER–RER) biosynthesis [1,30]. Similar to mitochondrial proteins, ER-resident proteins need to be directed to the ER. Transmembrane proteins undergo a cotranslational process here; that is, they are imported into the ER membrane at the same time that their mRNA is translated by the ribosome, thereby preventing exposure of hydrophobic regions and misfolding of proteins. This process is also facilitated by chaperones. Protein-translating ribosomes are attached directly to the ER, giving the ER the rough appearance observed by transmission electron microscopy (TEM), from which its name, the rough ER, is derived. Due to the need for ribosomal binding to the ER membrane to prevent transmembrane protein misfolding, more than 20 proteins enable their attachment exclusively in the RER, not in the SER [1]. At the SER, ER exit sites can be found, where transport vesicles carrying synthesized proteins and lipids bud off and go to their target region/organelle. The SER is also critical for the synthesis of steroid hormones, detoxification of water-insoluble drugs and storage of Ca^2+^ (further described in the next sections) [1].

### 2.3. The Ultrastructure and Tethering Proteins of MERCS

Although at first glance these two organelles seem to be functionally and structurally very different, they are physically and biochemically interconnected via MERCS. However, we still do not know the complete MERCS proteome or how certain proteins affect the ultrastructure and function of MERCS. To identify these players, researchers started to look at the protein profiles of subcellular MAM-enriched fractions in different tissues under normal and stress conditions. In 2013, Poston and colleagues identified 1212 proteins in the MAM-enriched fraction derived from mouse brain and found that most of these proteins have been reported to have a role in mitochondrial function and OXPHOS [31]. Other independent studies have also been performed with rabbit skeletal muscle, in which 459 proteins were identified [32], and with mouse and human testes (2800 proteins), and a second study was performed with mouse brains (2500 proteins) [33]. Other studies have used a similar approach to look at variations between proteins in MERCS during viral infection [34], in diabetes [35] and in mice with caveolin-1 (a pivotal regulator of cholesterol and component of MERCS) knocked out [36]. Recently, Magalhães Rebelo and colleagues clustered the common proteins identified in these different studies and showed that approximately 650 proteins in mouse brain tissue were found in three of the other aforementioned independent studies [31,33,37], but only 18 of these proteins were commonly found in all the aforementioned studies involving the mouse brain, liver and testis [37], suggesting that the MERCS proteome might be tissue-specific. They also showed that approximately 1190 proteins were found in two different immortalised human liver cells [31,32,35]. These studies are relevant to the field since they identified thousands of possible candidates that may be involved in the regulation of the structure and function of MERCS. Although several proteins in MERCS have been identified, and their functions have been reported, most of these candidates identified by proteomics remain to be validated. Of the previously validated proteins identified as related to MERCS, some have been reported to act as scaffold proteins, either tethering or acting as negative regulators of ER and mitochondria juxtaposition, while others are involved in the regulation of different biological functions, and some have even been reported to have both structural and functional roles [12,26]. Some relevant examples of the already identified scaffold proteins for this review include mitofusin 1 (Mfn1), mitofusin 2 (Mfn2), vesicle-associated membrane protein-associated protein B (VAPB) and protein tyrosine phosphatase-interacting protein 51 (PTPIP51).

Mitofusins were first reported to have a role in MERCS in 2008 by De Brito and colleagues; in their study, while Mfn1 was found to be present only in the OMM, Mfn2 was found both in the ER and mitochondria, allowing tethering between the two organelles by either heterodimers (Mfn1–Mfn2) or homodimers (Mfn2–Mfn2) [38]. For many years, it was widely accepted that these proteins act as tethering pairs in MERCS, and modulation of Mfn2 levels has been extensively used as a tool to modulate MERCS [39,40,41]. However, this model has been questioned since, more recently, other publications have reported that Mfn2 acts, in fact, as a negative regulator of MERCS; i.e., knocking out or knocking down Mfn2 increases the connectivity between the ER and mitochondria and increases the amount of Ca^2+^ shuttled from the ER to mitochondria [41,42,43]. Although the scientific community has not reached a consensus about the exact role of Mfn2 in MERCS, we can agree that modulation of Mfn2 levels alters the ultrastructure and function of MERCS. However, notably, in publications where Mfn2 was cited as a modulator of MERCS, the ultrastructure and function of the MERCS were not necessarily assessed, leading the authors to extrapolate changes in MERCS based on previous publications (mostly assuming that Mfn2 is a tethering protein).

VAPB and PTPIP51 were first shown to be MERCS proteins and to affect mitochondrial Ca^2+^ in 2012 by De Vos and colleagues [44]. In contrast to Mfn2, the role of the VAPB and PTPIP51 pair in MERCS seems to be consistent among different publications, making the modulation of these proteins a promising way to alter MERCS [45,46,47]. Recently, the VAPB and PTPIP51 pair has been found in synapses, and synaptic activity stimulates their interaction, leading to an increase in MERCS [47]. A recent review was published by Shirokova and colleagues, who extensively describe the role of MERCS in synapses [48].

Several other proteins have been shown to affect the ultrastructure of MERCS; however, a discussion of these proteins is not within the scope of this review. Some examples include PDZD8 [49], transglutaminase type 2 (TG2) [50], phosphofurin acidic cluster sorting protein 2 (PACS-2) [51], B cell receptor-associated protein 31 (BAP31) and TOM40 [52] and mitoguardin (Miga) [53].

In recent years, the ultrastructure of MERCS has been widely evaluated by TEM by assessing when mitochondria and the ER are closer than a specific distance—the cleft distance. Usually, the cleft distance is set between 10 and 80 nanometres (nm), where 30 nm is commonly set as the largest distance for contacting membranes. However, some publications have categorized these contacts into close (<30 nm) and long-distance contacts (>30 nm) [23,54]. Although much information can be obtained on the ultrastructure of MERCS from an electron micrograph, researchers usually quantify the number of MERCS observed in addition to measuring the contact distances. A general consensus in the field suggests that an increased number of or longer MERCS lead to increased connectivity between the ER and mitochondria and therefore increase the function of MERCS. However, the distance between the two organelles has been largely neglected and may provide further information about the nature of MERCS. Recently, Giacomello and Pellegrini suggested that MERCS can be classified into different groups/types according to their functions, e.g., Ca^2+^-MERCS and autophagy-MERCS, challenging the previous idea that a set of MERCS performs several biological functions. The authors suggest that a particular set of MERCS might have a particular proteome and, therefore, a particular cleft distance between the two organelles in accordance with their function (i.e., Ca^2+^-MERCS have a closer contact distance to allow cation exchange (approximately 15 nm), while autophagy-MERCS have long-distance contacts to accommodate autophagosome biogenesis (approximately 50 nm)) [23]. Another relevant parameter that the field is trying to address is the duration of these contacts. MERCS are extremely dynamic and change upon stress or metabolic demand [23]; therefore, a short but long-period MERCS may be as “strong” as a long but short-period contact.

Nevertheless, different functions have been suggested to occur at MERCS, with some of them exclusive to this region. In addition, alterations in the ultrastructure of MERCS affect their biological functions, including Ca^2+^ transfer from the ER to mitochondria, autophagosome formation and the unfolded protein response (UPR).

### 2.4. Ca^2+^ Shuttling from the ER to Mitochondria

Ca^2+^ transfer from the ER to mitochondria is one of the best-characterized functions of MERCS. Ca^2+^ is one of the major cellular secondary messengers, and even small variations in its concentration can lead to drastic alterations in cell homeostasis. Therefore, there is a need to buffer Ca^2+^ inside organelles (e.g., ER and mitochondria) to maintain low levels. This regulated buffering of Ca^2+^ allows the formation of Ca^2+^ “hotspots” that, upon stimulation, lead to a spatial–temporal release of these cations (further details about Ca^2+^ as a second messenger and Ca^2+^ homeostasis can be found in Berridge et al. and Bravo-Sagua et al. [55,56]). The majority of Ca^2+^ enters the ER via the sarco/endoplasmic reticulum Ca^2+^-ATPase (SERCA) pump and is released by either ryanodine receptors or inositol 1,4,5-trisphosphate receptors (IP3Rs) [57]. Together with glucose-regulated protein 75 (Grp75) and voltage-dependent anion-selective channel protein 1 (VDAC1), IP3Rs form a protein complex (IP3Rs-Grp75-VDAC1) that allows the passage of Ca^2+^ directly from the ER to the mitochondrial IMS. Although three different isoforms of IP3Rs have been identified (IP3R1, IP3R2 and IP3R3), the field has widely focused on the role of isoforms 1 (IP3R1) and 3 (IP3R3), probably because these two isoforms were the first described to be highly enriched in the MAM [58,59,60]. However, new evidence has recently shown that IP3R2 also plays a role in shuttling Ca^2+^ at MERCS [14,61]. Similarly, other isoforms of VDAC1, such as VDAC2, have recently been reported to be involved in functions related to MERCS [62]. However, the specificity of these isoforms in different types of MERCS or tissues remains to be uncovered. A few other proteins have been reported to modulate IP3Rs-Grp75-VDAC1, including Sigma-1 receptor (Sigma-1R) (which stabilizes IP3R3 in the MAM, thereby prolonging the Ca^2+^ signalling between the ER and mitochondria) [63] and TOM70 (the knockdown of which leads to misplacement of IP3R3 outside MERCS, and therefore to reduced Ca^2+^ shuttling from the ER to mitochondria) [64].

Ca^2+^ cannot diffuse through the impermeable IMM, and therefore, it enters the mitochondrial matrix via the mitochondrial calcium uniporter (MCU) complex. The MCU complex is formed by several regulatory proteins (mitochondrial calcium-uptake protein 1 (MICU1) and 2 (MICU2), essential MCU regulator (EMRE) and MCU paralogue (MCUb)) and by the MCU channel [65]. Surprisingly, MCU has a very low affinity for Ca^2+^, which prevents the uptake of Ca^2+^ into mitochondria when its level is low in the cytosol, and therefore, the level is also low in the IMS [66]. This condition limits Ca^2+^ uptake to only “hotspot” areas, such as MERCS where higher concentrations of Ca^2+^ overcome the low affinity of the MCU complex for Ca^2+^ [67].

In the matrix, Ca^2+^ can affect mitochondrial function differentially. Ca^2+^ can boost ATP production by activating pyruvate dehydrogenase [68,69,70], α-ketoglutarate dehydrogenase [71] and isocitrate dehydrogenase [72] in the TCA cycle. However, in excess, Ca^2+^ can lead to apoptosis by sensitizing mitochondria, lowering the threshold for mitochondrial permeability transition pore opening and activating the caspase-dependent mitochondrial pathway (a more detailed and comprehensive explanation of this mechanism can be found in Bravo-Sagua et al. [73]). In fact, an increase in MERCS and therefore higher Ca^2+^ flow into mitochondria have been shown to lead to the apoptosis of RBL-2H3 cells and dopaminergic neurons [74,75]. Therefore, excess Ca^2+^ needs to be extruded from mitochondria via the mitochondrial sodium/Ca^2+^ exchanger (NCLX) and taken up by the ER through the SERCA pump [76]. Thus, it is understandable that the levels of Ca^2+^, including the level inside mitochondria, must be tightly regulated since their imbalance can have an antithetical effect.

### 2.5. Autophagosome Formation

Macroautophagy, commonly known as autophagy, is a cellular process where specific targeted cargo is engulfed by an autophagosome. This autophagosome then fuses with a lysosome, forming an autophagolysosome, resulting in the degradation of the cargo and formation of macromolecule monomers that can be repurposed. Therefore, autophagy is known as a biological recycling process and is essential for cell homeostasis and development. Autophagy is usually activated during stress and is tightly regulated since its function must be integrated into responses to different insults [77,78].

Autophagosome formation can be marked by three phases: initiation, nucleation and expansion. One of the best-described pathways that leads to autophagosome formation is that of the energy sensor adenosine monophosphate (AMP)-activated protein kinase (AMPK) and mammalian target of rapamycin complex 1 (mTORC1). Under normal conditions, mTORC1 is active and promotes cell growth and anabolic metabolism, blocking autophagy. During starvation, when the levels of ATP decrease, AMPK is activated and induces catabolic metabolism by phosphorylating and inhibiting mTORC1. This leads to activation of the Unc-51-like autophagy-activating kinase 1 (ULK1) complex (which includes focal adhesion kinase family integrating protein (FIP200)), leading to the formation of an isolation membrane via the Beclin1–class III phosphatidylinositol 3-kinase (PI3KC3) complex. Next, maturation of the isolation membrane steps include several autophagy-related (ATG) proteins, and in the final steps, microtubule-associated protein 1A/1B-light chain 3 (LC3). LC3 is synthesised in an unprocessed form, cleaved at its C-terminus into LC3-I and then conjugated to PE, forming LC3-II. LC3-II has been widely used as a proxy for discerning mature autophagosomes since, in contrast to most other proteins involved in autophagosome formation, LC3-II does not dissociate from the autophagosomal membrane before its closure (a more detailed review on autophagosome formation and maturation was written by Lamb et al. and Grasso et al. [77,78]).

Even though the molecular mechanisms behind autophagosome formation and maturation are largely known, the exact place from which the isolation membrane originates remains puzzling. Since autophagosomes must form quickly in response to different inputs and stresses, it is believed that the isolation membrane must originate from organelles that are able to rapidly mobilize substantial amounts of membrane, such as the Golgi or ER. In fact, there are data showing that the isolation membrane can originate in the Golgi [79,80], plasma membrane [81], mitochondria [82] and MERCS [39,83]. MERCS were first shown to be among the places where the isolation membrane arises in 2013, when Hamasaki and colleagues showed that upon starvation, ATG5, ATG14 and double FYVE domain-containing protein 1 (DFCP1) were enriched in subcellular fractions enriched with MAM in mammalian cells. Furthermore, they showed that upon knockdown of PACS2 and Mfn2, the levels of ATG14 and DFCP1 in the enriched MAM fraction and the levels of LC3-II decreased, suggesting a diminished autophagosome formation [39]. Importantly, the ultrastructure of the MERCS was not assessed in this publication, and Mfn2 was assumed to be a tethering protein and, therefore, Mfn2 knockdown led to decrease in the connectivity between ER and mitochondria, leading to a decrease of autophagosome formation. However, as mentioned above, recent publications have suggested that Mfn2 has a negative role in the regulation of these contacts. In fact, it was recently shown that during starvation, the number of MERCS and the mitochondrial function are upregulated immediately before autophagosome formation. However, when the levels of LC3-II increase, mitochondria and ER juxtaposition decrease at the same time point, together with a decrease in mitochondrial function and an increase in the levels of Mfn2 [84]. In addition, Gomez-Suaga and colleagues showed that knockdown of VAPB or its partner PTPIP51 led to a decrease in MERCS and an increase in basal autophagy and autophagic flux. Accordingly, the overexpression of these proteins led to an increase in the juxtaposition between the ER and mitochondria and a decrease in basal autophagy and autophagic flux. Surprisingly, overexpression of VAPB or PTPIP51 prevented the formation of autophagosomes after rapamycin and torin-1 (inducers of autophagy) treatment but not starvation [46]. In summary, these data suggest that connectivity between the ER and mitochondria is negatively correlated with autophagosome formation, even though this negative effect is dependent on the nature of the autophagy stimulus. One of the possible mechanisms that explains how an increase in MERCS can lead to a decrease in autophagosome formation involves Ca^2+^. Gomez-Suaga and colleagues showed that the aforementioned changes are associated with Ca^2+^ shuttling from the ER to mitochondria via IP3Rs since blocking IP3Rs with Xestospongin C or the MCU complex with ruthenium-360 abrogated the effect of VAPB and PTPIP51 overexpression on autophagosome formation [46]. However, Ca^2+^ signalling has been shown to affect autophagy in both positive and negative ways. For example, since mitochondrial Ca^2+^ influences ATP production and because AMPK is regulated by the AMP:ATP ratio, it is easy to understand that a decrease in Ca^2+^ in the mitochondria results in a decrease in ATP and therefore induces activation of AMPK and autophagy. Hence, it is not surprising that a genetically induced decrease in IP3Rs, such as by knockdown, or the inhibition of their activity with Xestospongin B treatment led to activation of autophagy via activation of AMPK under fed conditions [85]. However, under starvation conditions, treatment with Xestospongin B actually leads to the inhibition of autophagy [86]. To further complicate our understanding of autophagosome origin, ATP has also been shown to be upregulated during amino-acid starvation in trypanosomes and to be essential for certain autophagosome-assembly steps [87]. Further studies are required to better understand the exact role of Ca^2+^ in autophagy regulation, but the differences in results are probably connected with metabolic status and energy availability under fed versus starved conditions.

### 2.6. The Unfolded Protein Response in the ER

Due to the major role of the ER and mitochondria in the cell, it is not surprising that these organelles have developed signalling pathways that ensure their functionality during stress, such as that caused by an accumulation of misfolded proteins. Protein homeostasis results in a balance between the accumulation of unfolded proteins and the folding capacity of the cellular system. The unfolded protein response (UPR) is a conserved adaptive pathway that allows the recovery of ER (UPR^ER^) and mitochondria (UPR^mt^) to their normal functions even upon the accumulation of misfolded proteins in these organelles. Although these processes can be activated by several pathways, the general mechanism consists of halting protein synthesis (except for chaperones) to decrease the burden of protein misfolding. However, sustained and prolonged conditions can also have negative effects, including apoptosis [88,89].

The UPR^ER^ consists of three integrated signalling pathways activated by activating transcription factor 6 (ATF6), protein kinase RNA-like endoplasmic reticulum kinase (PERK) or inositol-requiring enzyme 1 (IRE1). These three proteins, under normal conditions, are inhibited by a direct interaction with immunoglobulin heavy-chain-binding protein (BiP). Upon stress stimulation, such as the accumulation of misfolded proteins, BiP releases the sensor proteins, thereby inducing the activation of the UPR^ER^. In particular, when dissociated from BiP, PERK forms stable homodimers via its luminal domains, which trans-phosphorylate each other’s cytosolic kinase domain. Tetramers of PERK can also be found and are believed to have an increased state of activation [90]. Activated PERK will then phosphorylate the α-subunit of eukaryotic initiation factor 2 (eIF2α), resulting in the activation of the stress-responsive activating transcription factor ATF4. Altogether, the activation of these two factors leads to the inhibition of ribosomal translation initiation and a shift to the increased production of stress-responsive proteins. ATF4 can induce the expression of C/EBP homologous protein (CHOP), which, in turn, induces stress-responsive genes such as ER oxidase 1 (*ERO1*), protein phosphatase 1 (*PP1)* and PP1 cofactor DNA damage-inducible protein 34 (*GADD34*), which dephosphorylate eIF2α, deactivating the whole pathway. A more detailed review of UPR^ER^ was described by Santos et al. and Rainbolt et al. [88,91].

The possible involvement of MERCS in ER stress arises from the fact that ER stress can be transmitted to mitochondria via changes in the transfer of metabolites, such as Ca^2+^, between the two organelles. In fact, MERCS is increased in the early phases of ER stress, leading to an increase in Ca^2+^ inside mitochondria and in ATP levels. This increase raises the energy levels in the cell, helping it cope with the ER stress response [92]. On the other hand, chronic exposure to ER stress leads to an overload of Ca^2+^ in efflux from the ER. However, as mentioned in Section 2.4, when this overflow of Ca^2+^ into mitochondria becomes excessive and overwhelming, this results in apoptosis and programmed cell death [73,91,92]. The induction of ER stress by tunicamycin was recently shown to decrease MERCS via disruption of the BAP31–TOM40 tethering complex [52]. In particular, PERK has also been shown to affect mitochondrial function. Cells deficient in PERK manifest increased basal and maximal respiration and an increase in ROS, impaired mtDNA biogenesis and altered apoptosis [91]. Moreover, PERK has been shown to localize to MERCS and is required for proper coupling of the ER and mitochondria and for ROS-induced apoptosis. In addition, depletion of PERK leads to fewer MERCS [93]. PERK activation has also been reported to facilitate mitochondrial proteostasis by modulating protease Lon during ER stress, preventing mitochondrial dysfunction during ER stress [91] and by increasing the levels of the Grp75 protein (also known as heat shock protein (HSP) 70 ATP-dependent chaperone HSPA9 or mortalin) in MERCS. Overexpression of Grp75 attenuates ROS levels in models upon glucose deprivation, in which ER stress is activated [94] and attenuates the cell toxicity induced by the amyloid β-peptide (Aβ) [95,96]. Additionally, dysregulation of normal mitochondrial function and dynamics by deletion of Mfn2 leads to the activation of UPR^ER^ via PERK, and if PERK is depleted in these cells, mitochondrial dysfunction is attenuated. Unfortunately, the ultrastructure of the MERCS was not assessed in this publication. In the same publication, the authors also showed that Mfn2 interacts with PERK and negatively regulates its function [97]. In addition, knocking down PACS-2, and therefore alterations in MERCS ultrastructure and Ca^2+^ function, leads to activation of BiP and the UPR^ER^ [51]. Additionally, IRE1 has been found in MAM and is stabilized by the Sigma-1R protein in MERCS [98]. In summary, these data suggest a tight relationship between ER stress, MERCS and mitochondrial ultrastructure and function.

### 2.7. The Unfolded Protein Response in Mitochondria and Mitochondrial Quality Control

Mitochondria have a series of pathways that evolved to maintain their homeostasis, called mitochondrial quality control (MQC). MQC can be considered a series of biological processes that attenuate mitochondrial damage or stress and, when irreversible, lead to the destruction of the damaged part of the mitochondrial network. Upon protein aggregation in mitochondria, one of the first responses of the MQC is a mitochondrial integrated stress response that leads to the activation of the UPR^mt^ by “retrograde signalling” to the nucleus [99]. Similar to the UPR^ER^, the activation of the UPR^mt^ leads to the attenuation of protein translation in mitochondria and an increase in the production of nuclear-encoded chaperones (e.g., Hsp-60 and Grp75 (and its orthologue Hsp-6 in *Caenorhabditis elegans—C. elegans*)) and proteases (e.g., ATP-dependent caseinolytic protease proteolytic subunit (ClpP) and Lon)) [89,100,101]. In *C. elegans*, activating transcription factor associated with stress-1 (ATFS-1) has a mitochondrial and nuclear targeting sequence and is essential for the activation of the UPR^mt^. Under normal conditions, this protein is imported into mitochondria, where it can be degraded by Lon [89]. During mitochondrial stress, ATFS-1 localizes to the nucleus, where it acts as a transcription factor and activates the expression of chaperones, proteases and other UPR^mt^ pathways. In addition, during the UPR^mt^, ATFS-1 can accumulate in the matrix where it binds to mtDNA, inhibiting transcription [100,101]. Recently, the mammalian orthologue of ATFS-1 was identified: ATF5 [102]. Since “retrograde signalling” can be activated by different mechanisms, including changes in AMP/ATP ratios, ΔΨ_m_, Ca^2+^ homeostasis and ROS levels [103], and because all these biological processes have been shown to be regulated by MERCS, it is not surprising that MERCS may have a role in the modulation of MQC and the UPR^mt^. In fact, one of the few studies that examined this connection showed that, during mitochondrial stress, eIF2α is phosphorylated by general control nonderepressible-2 (GCN-2, one of the eIF2α kinases that is active during amino-acid starvation) in a ROS-dependent manner, attenuating protein synthesis and activating the UPR^ER^ [104]. A series of other studies showed that PD fly models show impaired mitochondrial function and increased ER and mitochondrial stress, characterized by increased levels of BiP and phosphorylated eIF2α, dependent on PERK. These animal models also showed an increase in MERCS that was restored upon the downregulation of *Drosophila melanogaster* (*D. melanogaster*) mitofusin (dMfn) [105]. Knocking down ATF4 downstream targets serine hydroxymethyltransferase 2 (Shmt2) and mitochondrial NAD-dependent methylenetetrahydrofolate dehydrogenase-cyclohydrolase (Nmdmc) leads to mitochondrial fragmentation and loss of ΔΨ_m_, and suppressing the upregulation of these targets in the same PD fly models worsened their phenotype, while overexpression of these targets improved the model phenotype. [106]. Knocking down dMfn led to an increase in ATF4, Shmt2 and Nmdmc, and the overexpression of Nmdmc recovered the phenotype of dMFn RNAi-treated flies, decreasing the levels of ATF4 [107]. Interestingly, ATF4 has also been shown to be activated upon mitochondrial stress induction [108]. Additionally, loss of Mfn2 has also been shown to activate ER stress [109]. In summary, these data suggest that, similar to their roles in UPR^ER^, eIF2α and ATF4 seem to play roles during mitochondrial stress. However, further studies need to be performed to increase the understanding of the role of MERCS in the UPR^mt^ and of how mitochondria communicate with the ER.

When the UPR^mt^ is overwhelmed, mitochondria employ a second mechanism to cope with stress. Mitochondria are extremely dynamic organelles and undergo cycles of fusion and fission to maintain the health and function of their extensive network. These alterations between cycles allow mitochondria to undergo specific functions, e.g., dispose of damaged mtDNA and/or proteins by segregating the damaged components into daughter mitochondria via mitochondrial fission. Mitochondrial fusion is regulated by three major GTPase proteins: optic atrophy 1 (OPA1) in the IMM and Mfn1 and Mfn2 in the OMM. OPA1 anchors to the IMM via its N-terminus, while the C-terminal GTPase domain faces the IMS and is believed to be critical for the fusion between two mitochondria [110]. Mfn1 and Mfn2 are structurally similar to each other; although Mfn1 is involved in mitochondrial docking and fusion, Mfn2 has lower GTPase activity and therefore stabilizes the interactions between two mitochondria [111]. Embryonic ablation of either of these proteins is lethal [112]. Mitochondrial fission is also regulated by several proteins, with the GTPase dynamin-related protein 1 (DRP1) being one of the best-characterized. DRP1 is a cytosolic protein that can be recruited to mitochondria where it oligomerizes into a ring-like structure, leading to membrane strangling and ultimately to mitochondrial fission. Different adaptor proteins have been shown to recruit DRP1 into mitochondria and regulate its GTPase activity according to different cellular responses and energy states [113]. Due to the particular anatomy of neuronal cells; i.e., with projections that can be extremely long, the maintenance of mitochondrial dynamics is extremely important to ensure proper cellular distribution and function of this organelle. In AD and other NDs, it is believed that an imbalance between fission and fusion leads to increasingly fragmented and progressively less-functional mitochondria [114,115].

The final MQC mechanism involves the recycling of daughter mitochondria using the autophagy machinery to undergo mitophagy. Mitophagy ensures the elimination of impaired mitochondria that have been separated from the mitochondrial network by fission. Mitochondrial biogenesis can then occur through healthy mitochondria to replace cleared defective organelles [116,117]. Under normal conditions, phosphatase and tensin homologue-induced kinase 1 (PINK1) is imported into mitochondria via the TOM and TIM complexes. In the IMM, PINK1 is cleaved by presenilin-associated rhomboid-like protein (PARL) and matrix-processing peptide (MPP) [116,117,118,119]. Since mitochondrial import via IMM is ΔΨ_m_-dependent, when mitochondria are dysfunctional, ΔΨ_m_ is lost, preventing the import to or through the IMM [120]. In this situation, PINK1 is not cleaved by PARL and MPP and will accumulate in the OMM with the TOM complex. PINK1 can phosphorylate different proteins, including Mfn2, leading to the recruitment of Parkin to depolarized mitochondria. Parkin is an E3 ubiquitin ligase that ubiquitinates several proteins in the OMM (including the VDAC1 [121], TOM70 [122], Mfn1 [123] and Mfn2 [124] proteins in MERCS), leading to the activation of mitophagy. This action involves the recruitment of autophagosome proteins and results in the engulfment of damaged mitochondria by the mitophagosome. Other mechanisms have been shown to induce mitophagy [116,117]. Similar to autophagosomes, MERCS have also been reported to be involved in mitophagosome origination [125,126].

Mitochondrial fission has been shown to facilitate mitophagy [127]. Interestingly, the ER has been reported to surround mitochondria where the fission site will occur, forming MERCS. This allows the ER protein inverted formin 2 (INF2) and the mitochondrial protein actin-nucleating Spire and Arp2/3 complexes to recruit actin–myosin assembles, which, together with Drp1, induce mitochondrial fission [128,129,130,131]. More recently, a study showed that Drp1 is associated with the ER during the mitochondrial fission process, tubulating the ER and facilitating its interaction with mitochondria [132]. Moreover, this process seems to be regulated by mechanisms in the mitochondrial matrix, since actively replicating mtDNA is present in these ER-associated mitochondrial constriction and division sites, suggesting coordination between mtDNA synthesis and mitochondrial division [133,134]. However, it was recently shown that under stress, mitochondrial fragments colocalized with LC3 in Drp1-knockout yeast cells [135], suggesting that other mechanisms are involved in mitochondrial fission during mitophagy. Furthermore, ablation of Mfn2 in different cell types has also been reported to impair mitophagy, showing that mitochondrial fusion is also important during this process, since it helps the interaction between PINK1 and Parkin [124,136]. Altogether, these data indicate that MERCS can influence mitophagy by modulating mitochondrial dynamics, in addition to its obvious role in modulating the formation of the isolation membrane. In fact, it was recently shown that mitochondrial fusion is regulated at MERCS and that mitochondrial fusion and fission are spatially coordinated at this subcellular localization [137].

In summary, these data suggest that mitochondria and the ER communicate under stress conditions and mutually support each other to maintain homeostasis. However, several of the mechanisms that ensure this communication under stress remain unknown, as does the exact role of MERCS in this process.

### 2.8. Other Functions of MERCS

Other functions of MERCS have been described. ROS production and clearance is a very fine-tuned process in a cell. While ROS are important secondary messengers, high levels of these unstable molecules can damage DNA and proteins, leading to oxidative stress in the cell. ROS are mostly produced as by-products of OXPHOS and can be cleared by antioxidant enzymes, such as superoxide dismutases (SODs). With age and in disease, this balance is thought to be lost, leading to an increase in ROS [138,139]. Recently, MERCS have been shown to control ROS nanodomains. During Ca^2+^ transfer from the ER to mitochondria, ROS (in the form of H_2_O_2_) were immobilized in the space between the ER and mitochondria, which allowed sustained Ca^2+^ oscillation [140]. In addition, an increase in the number of contacts formed between the ER and mitochondria led to an increase in ROS in ex vivo *D. melanogaster* [141]. Interestingly, NADPH oxidase 4, one of the multi-subunit enzymes of the ETC, was shown to localize at MERCS and protect cells against Ca^2+^-induced cell death by inhibiting IP3R via phosphorylation [142]. A more detailed review on the interplay between MERCS and ROS was recently published by Fan and colleagues [143].

Another widely described biological process at MERCS is phospholipid and cholesterol formation and metabolism. Phospholipids are polar molecules with long hydrophobic tails. Therefore, they cannot be transported through aqueous phases, such as the cytosol, and need to be transported in vesicles or trafficked directly between phospholipidic layers. Several proteins involved in lipid and phospholipid metabolism have been described to be involved in MERCS. A common example of phospholipid metabolism at MERCS is the conversion of PSer to PE. PSer is transported from the ER to the IMM, where PSer decarboxylase converts it into PE. PE is then transported back to the ER, where it can be converted to PC by PE N-methyltransferase. Jean Vance has written two reviews in which this process is described in greater detail [144,145].

Due to the aforementioned vital roles of MERCS-related biological processes, it is not surprising that alterations in MERCS, proteins related to MERCS and functions of MERCS have been associated with several different types of diseases, including ND, cancer, diabetes, obesity and viral infectivity [12,13]. In the subsequent sections, we focus on the roles of MERCS in different NDs with a focus on AD.

## 3. MERCS in Alzheimer’s Disease

### 3.1. Alzheimer’s Disease and the Mitochondrial Cascade Hypothesis

AD is the most common form of dementia in the world, and it is believed that 25 to 35 million people suffer from this pathology worldwide. AD is a complex multifactorial disorder in which patients present cognitive decline, loss of memory, behavioural changes and, in terminal phases, full dependency and the need for full-time caregivers. This disease is characterized by progressive loss of neuronal cells (mostly cholinergic neurons in the forebrain and glutamatergic neurons in cortical areas and the hippocampus) and by the accumulation of intracellular neurofibrillary tangles (NFT, constituted by hyperphosphorylated tau protein) and extracellular amyloid plaques (constituted by Aβ) [146,147]. To date, the accumulation of intracellular Aβ is believed to be the cause of neurodegeneration in AD [148,149]. AD can be classified into an idiopathic form, sporadic AD (SAD), and a hereditary form, familial AD (FAD). Even though drugs such as acetylcholinesterase inhibitors (e.g., memantine) slow cognitive decline, there are still no drugs that halt the ongoing neurodegeneration [146].

One can argue that one of the major reasons for the dearth of effective drugs in AD is that the exact molecular and cellular mechanism underlying the aetiology of AD remain unknown. In fact, several cellular processes have been reported to be altered in AD, including oxidative stress, mitochondrial dysfunction, alterations in cholesterol metabolism, inflammation and activation of the UPR^ER^ and UPR^mt^ [150,151,152]. Due to the pivotal role of mitochondria in the cell, it is not surprising that alterations in normal mitochondrial functioning or structure can have a major impact on the cell and lead to different human pathologies. In fact, increased mitochondrial fragmentation and ROS, as well as decreased activity of TCA cycle enzymes, OXPHOS and ATP production, have been shown in AD patients and AD models. Some of these events are evident even before plaque formation, suggesting that mitochondrial dysfunction precedes activation of the amyloidogenic pathway [153]. In addition, different groups have shown that Aβ is present in the OMM and can be imported into mitochondria via the TOM complex and receptor for advanced glycation end products (RAGE) and therefore is found inside mitochondria in postmortem AD brains [154,155,156,157,158,159]. Due to the accumulation and aggregation of Aβ in mitochondria, as well as impaired mitochondrial function, it is not surprising that the UPR^mt^ was also found to be upregulated in FAD and SAD [160,161], in cells overexpressing APP and in AD mouse models [161], as manifested by the increased levels of Hsp-60, Grp75, ClpP and Lon. *C. elegans* overexpressing Aβ also showed an increase in the UPR^mt^ and mitophagy, and ATFS-1 depletion in these worms led to impaired mitochondrial function, a reduction in the UPR^mt^, mitophagy and increased accumulation of Aβ and paralysis. However, induction of the UPR^mt^ by overexpressing ATFS-1, silencing mitochondrial ribosomal protein mrps-5 or inhibiting mitochondrial translation with doxycycline increased mitochondrial fitness and decreased Aβ accumulation [161]. Notably, the UPR^ER^ has also been shown to be upregulated in AD. BiP levels and phosphorylated PERK and eIF2α have also been shown to be increased in postmortem AD brains and in models with increased Aβ. In fact, attenuating the increase in the levels of PERK and phosphorylated eIF2α alleviated the AD-like phenotypes in a transgenic AD model [162,163].

In summary, these data led to the postulation of a mitochondrial cascade in AD, where mitochondria mediate or even initialize the pathology (a more detailed review of the mitochondrial cascade hypothesis in AD was recently published by Swerdlow [153]). However, neither the OMM nor mitochondria themselves possess the biochemical milieu required for Aβ production (i.e., OMM does not have lipid rafts, and the mitochondria pH is not acidic), and mitochondrial dysfunction cannot explain some of the dysfunction observed in AD, e.g., alterations in phospholipid and cholesterol metabolism and general Ca^2+^ dysfunction. Only in the past 10 years has it been possible to understand how Aβ formation can occur in close proximity to mitochondria and why the aforementioned biological processes are altered due to MERCS, allowing the emergence of the MERCS hypothesis in Alzheimer’s disease.

### 3.2. The Role of MERCS in Aβ Production

As mentioned before, Aβ is the major component of amyloid plaques, and one of the fragments originates from the successive cleavage of amyloid precursor protein (APP) by β- and γ-secretase [164]. In the amyloidogenic pathway, APP is first cleaved by β-secretase, forming a soluble APPβ fragment and C99. C99 is further cleaved by γ-secretase, forming the APP intracellular domain (AICD) and Aβ. γ-secretase is composed of four different proteins, in which presenilin 1 or 2 (PS1 or PS2) correspond to the catalytic core protein of this protein complex [165,166]. Mutations in APP, PS1 or PS2 have been shown to cause FAD [147,167]. Interestingly, APP and γ-secretase have been detected in different subcellular localizations, including the ER, lipid rafts in the plasma membrane and mitochondria [155,168]. Area-Gomez and colleagues showed, for the first time, that PS1, PS2 and APP were enriched and active in a subcellular fraction enriched with MAM [169] and that this fraction behaved similar to a lipid raft since it was resistant to detergent [54]. Moreover, mouse embryonic fibroblasts (MEFs) lacking PS1 and PS2, as well as fibroblasts derived from AD patients, showed increased connectivity between the ER and mitochondria [54]. In addition to this study, Schreiner and colleagues showed that Aβ is formed in this MAM-enriched fraction (but not in fractions enriched with pure mitochondria), and upon overnight incubation with the γ-secretase inhibitor L-685,458, Aβ production was significantly decreased, showing that not only is Aβ present in this fraction, but it can also be formed here [170]. In 2017, Del Prete and colleagues further corroborated these findings by showing that APP, Aβ, β- and γ-secretase are present and active in the MAM fraction derived from SH-SY5Y and mouse brains [171].

To further advance the role of MERCS in Aβ production, a few publications have shown that modulation of MERCS influences Aβ levels and formation. A stable Mfn2-knockout MEF cell line showed a decrease in the APP fragment AICD and accumulation of C99, suggesting an impairment of γ-secretase cleavage [54]. These data were further confirmed since acute knockdown of Mfn2 in HEK293 cells overexpressing APP with the Swedish mutation (APP^Swe^)—one of the mutations that causes FAD by leading to increased levels of Aβ40 and Aβ42 [172]—led to an increase in connectivity between the ER and mitochondria and a decrease in Aβ production due to impaired γ-secretase maturation and therefore a decrease in its activity [41]. Altogether, these data show that APP, β- and γ-secretase are present at MERCS, that Aβ can be formed at this subcellular region and that modulation of MERCS affects Aβ production. However, it is still unknown exactly how this process is realized at MERCS, since APP and γ-secretase must mature in the Golgi apparatus and/or endosomes [173,174]. Therefore, several hypotheses have been postulated, including that APP and γ-secretase return to MERCS after their respective maturation. In fact, it has been shown that endosomes are found to contact mitochondria and that the retrieval receptor Rer1p can transport active γ-secretase from the Golgi to the ER [175,176,177]. A new study supporting the production of Aβ in MERCS demonstrated that the majority of Aβ is produced by a supercomplex formed by β- and γ-secretase, which localizes in the perinuclear region of the cell, where the ER and mitochondria are known to be abundant [178]. However, whether this supercomplex exists at the MERCS remains unknown. Further work needs to be performed to better understand how mature APP and γ-secretase return to MERCS and how Aβ production is regulated at MERCS.

### 3.3. The Effect of Aβ on the Ultrastructure and Function of MERCS

For several years, it was believed that amyloid plaques were critical for the neurodegeneration observed in AD. However, the presence of extracellular plaques does not explain how they lead to cell degeneration, and the number of plaques does not correlate with the cognitive decline found in AD patients [148,149]. Recently, oligomeric forms of Aβ; namely, Aβ42, were shown to be the most toxic forms of Aβ and to correlate with the cognitive decline found in AD. However, there is still no consensus within the scientific community regarding the exact mechanisms that lead to cell failure and death [148,149]. In fact, when Leal and colleagues assessed the ultrastructure of MERCS in brain biopsy samples derived from patients with idiopathic normal pressure hydrocephalus (iNPH), they observed no significant differences between biopsies with or without staining for extracellular amyloid plaque [179]. These data suggest that the accumulation of amyloid plaques does not affect the ultrastructure of MERCS. However, a positive correlation between the number of MERCS and the ventricular levels of Aβ42 was found in the same patients, suggesting that the monomeric and/or oligomeric form of Aβ42 might affect the ultrastructure of MERCS. These data are further supported by different publications. In 2013, Hedskog and colleagues showed that incubation of mouse primary cortical neurons with conditioned medium derived from CHO cells overexpressing APP with the Indiana mutation (APPV717F) (which increases the Aβ42/Aβ40 ratio) led to an increase in the proximity between the VDAC1 and IP3R3 proteins in MERCS, as measured by proximity ligation assay (PLA). An increase in Ca^2+^ shuttling from the ER to mitochondria was also reported in SH-SY5Y cells upon treatment with this medium under the same conditions [180]. These data were used as a proxy for an increase in MERCS, and therefore, the authors concluded that Aβ increases the connectivity between the ER and mitochondria. However, two of the major drawbacks of this study were the lack of assessment of MERCS by methods in addition to the PLA of one protein pair specific to MERCS, as well as the fact that the authors did not identify the component in the conditioned medium that led to the alterations in MERCS (i.e., the type of Aβ and/or whether it was in aggregation form). In 2017, Del Prete showed that overexpression of APP^Swe^ in SH-SY5Y cells led to an increase in the connectivity between the ER and mitochondria and an increase in the number of lipid droplets, which have been shown to interact with MERCS [9,171]. Although the authors showed that incubation of WT SH-5YSY cells with oligomeric Aβ42 increased the number of lipid droplets, they did not assess alterations in the ultrastructure of the MERCS. In fact, the model used overexpressed APP, increasing the levels of not only Aβ but also different catabolites derived from APP cleavage, including C99, in the same publication [171]. Therefore, the increase in MERCS observed by the authors cannot be said to be caused exclusively by Aβ. In fact, in the same year, Pera and colleagues showed that inhibition of γ-secretase led to an enrichment of C99 in the MAM fraction, resulting in an increase in the connectivity between the ER and mitochondria, affecting the amount of lipid-droplet formation [181]. Further details on the alterations in cholesterol and phospholipid metabolism in AD can be found in a recent review published by Agrawal and colleagues [182]. However, a recent study showed that incubation of rat primary hippocampal neurons with oligomeric Aβ42 led to increased connectivity between the ER and mitochondria, increased Ca^2+^ transport from the ER to mitochondria and increased ROS and apoptosis rates [183]. This study thus supports previous studies that showed that oligomeric Aβ42 promotes the influx of extracellular Ca^2+^ by activating Ca^2+^-permeable channels and forming pores in the plasma membrane [184,185] and in the ER in an IP3R-dependent manner [186]. Similarly, it was recently shown that primary cortical neurons derived from the *App^NL-F^* knock-in mouse AD model, which presents higher levels of Aβ42 but not other APP fragments since it is a knock-in model [187], exhibit an increase in ER-mitochondria connections. Moreover, WT animals treated with synthetic monomeric and oligomeric Aβ42 showed a similar increase. In addition, when cells were treated with oligomeric Aβ42 and the oligomeric Aβ-neutralizing antibody fragment scFvA13, the increase in MERCS was abolished, showing that this increase is Aβ42-dependent [84]. In *D. melanogaster*, overexpression of Aβ42 leads to reduced climbing ability and a decreased lifespan. However, both phenotypes were recovered upon a genetic increase in MERCS [141], suggesting that the increase in MERCS observed in different AD models might act as a rescue mechanism to recover from Aβ stress. However, a recent study using Förster resonance energy transfer (FRET) live imaging in neurons derived from transgenic rats and overexpressing APP (and thus increasing Aβ and other APP fragments) showed a decrease in the number of lipid MERCS (with cleft distance of <10 nm), shorter MERCS and a decrease in mitochondrial respiration. No changes were observed in contacts with distances of 10–20 nm [188]. However, as mentioned before, use of overexpressing models or investigating a particular tethering pair at MERCS might not be ideal for estimating the overall alterations in the ultrastructure of MERCS in AD.

Recently, Leal and colleagues showed that *App^NL-F^* and *App^NL-G-F^* mice—which, similar to *App^NL-F^*, have increased levels of Aβ42 but not of other APP fragments [187]—also showed an increase in MERCS in CA1 (hippocampus) at the age of 10 months, but no difference was found in the cortex or in any of these brain regions at earlier ages [84]. These data suggest that although Aβ has an effect in increasing MERCS, this effect might not occur until later stages of the pathology. These data are further supported by Lau and colleagues, who showed that the VAPB and PTPIP51 pair as well as IP3R1 in MERCS are not altered in early Braak stages (III–IV) but are altered in later stages (Braak stage VI) [189]. In fact, it was also shown that there is a positive correlation between the number of MERCS and ageing [179], suggesting that MERCS also increase in “healthy” ageing. In addition, mice overexpressing APP with the Swedish mutation showed alterations in proteins related to MERCS at three months [190]; however, the ultrastructure of the MERCS was not assessed.

In summary, several studies in the field suggest that Aβ increases MERCS. Nevertheless, since the aforementioned studies are based on different models and different methodologies to assess MERCS, it is impossible to compare them and understand the discrepancy in the data. Further studies will allow us to understand whether these differences in the different publications arise from the different models and techniques used or because different types of MERCS were analysed. Additionally, whether these alterations in the ultrastructure and function of MERCS are caused directly by Aβ or by an indirect pathway remains unexplored.

### 3.4. The Effect of Tau on the Ultrastructure and Function of MERCS

In contrast to studies on Aβ, only a few studies regarding MERCS and tau have been published. In 2009, Perreault and colleagues showed that overexpression of human tau in mice leads to an increase in the number of contacts between mitochondria and the rough ER [191]. Recently, Cieri and colleagues showed that overexpression of WT tau (2N4R) and caspase 3-cleaved truncated tau protein (2N4RΔC20), which induces fibrillation and seeding of WT tau, led to the localization of these proteins into the IMS and OMM, as well as a decrease in the steady-state ER Ca^2+^ content in HeLa cells. They also showed that overexpression of 2N4RΔC20 in the same cells led to an increase in the short-range distance (8–10 nm), as measured by split-GFP-based sensors (SPLICS_S_) of MERCS, while the long-range (40–50 nm) sensor (SPLICS_L_) was not altered [192]. However, an ultrastructural study performed in brain biopsy samples obtained from iNPH patients showed that the presence of amyloid plaques and NFT was associated with decreased MERCS length (MERCS was defined as the distance between the ER and mitochondria ≤ 30 nm), while amyloid plaques did not alter the ultrastructure of MERCS. In addition, there was no correlation between the ventricular levels of tau and MERCS [179]. Other studies have assessed mitochondrial dysfunction in tau models, but MERCS were not assessed, and we can only postulate that MERCS were altered. For example, the overexpression of human tau disrupts mitochondrial function and mitochondrial dynamics, leading to organelle elongation and accumulation in the perinuclear region, whereas explained above, is highly enriched in the ER. Additionally, the same models show an increase in the levels of Mfn1 and Mfn2, which can have an effect on MERCS [193]. A more recent study showed that tau inhibits mitochondrial Ca^2+^ levels by affecting its efflux, but as in other studies, MERCS were not assessed [194]. Therefore, further studies are required to better understand how tau can affect the ultrastructure and function (or vice versa) of MERCS, as well as the mechanisms underlying it.

### 3.5. Alterations in the Ultrastructure and Function of MERCS in other AD-Related Models

One of the first pieces of evidence showing that MERCS can be altered in AD was based on the fact that SH-SY5Y cells overexpressing PS2 with the FAD T122R mutation showed increased Ca^2+^ transfer from the ER to mitochondria and closer juxtaposition between the ER and mitochondria compared to the cells overexpressing WT PS2 [195]. Similar data were obtained from primary cortical neurons derived from PS2 N141I mice [196]. Interestingly, mutations in PS1 have been reported to change [197] and not change MERCS [195]. Similarly, in *C. elegans*, a FAD-linked mutation in Sel-12 (orthologue of PS) leads to neurodegeneration and elevated mitochondrial Ca^2+^ content, which stimulates mitochondrial respiration, resulting in an increase in mitochondrial superoxide production. However, the ultrastructure of MERCS was not evaluated [198]. More recently, PS2, but not PS1, was shown to modulate MERCS, but only in the presence of Mfn2. In this study, the authors also showed that PS2 and Mfn2 physically interact, suggesting that this is the mechanism by which PS2 modulates MERCS [199]. Further details on the role of PS2 in AD and Ca^2+^ dysfunction can be found in [200].

Concerning lipid metabolism, the first alterations of MERCS in AD models were shown when there was an increase in the total levels of cholesterol, free cholesterol, cholesteryl esters, PSer, PE and lipid droplets in cell lines lacking either PS1, PS2 or both. In addition, fibroblasts obtained from SAD and FAD patients showed an increase in lipid-droplet formation. An assessment of the ultrastructure of the MERCS by colocalization and TEM showed that the aforementioned cell models and fibroblasts derived from AD patients had an increase in connectivity between the ER and mitochondria compared with the respective controls [54].

Autophagy was first shown to be impaired in AD by Nixon and colleagues when they observed the accumulation of APP, C99, Aβ and PS1 in immature autophagosomes, named autophagic vacuoles (AVs), in postmortem AD brains [201,202]. These results were believed to be associated with impaired fusion with the lysosome and therefore the elimination of Aβ. However, starvation of animals with a water-only diet was not enough to degrade Aβ, even though autophagy was activated in the retrosplenial dysgranular and cerebellar cortex [203]. Currently, we know that modulation of autophagy affects not only Aβ degradation, but also its production [202,204] and secretion [205]. Furthermore, pharmacological activation or inhibition of autophagy in SH-SY5Y neuroblastoma cells led to an increase in α-, β- and γ-secretase activity and extracellular Aβ42 levels, with higher levels upon inhibition [204]. In addition, PS1 and PS2 have also been shown to modulate autophagy [206,207]. Recently, it was reported that TOM70 is present at MERCS and plays a pivotal role in Ca^2+^ shuttling from the ER to mitochondria. Although knockdown of TOM70 did not change the ultrastructure of MERCS, it led to decreased IP3R3 at MERCS and therefore a decrease in complex IP3R3-Grp75 formation. Moreover, this decrease in TOM70 levels also led to a decrease in Ca^2+^ shuttling to the mitochondria, decreased ATP formation and an increase in autophagy [64]. Recently, TOM70, together with TOM40, was reported to be essential for autophagosome formation since it recruits ATG2A to MERCS during autophagosome formation [208]. In summary, these data, together with the fact that the isolation membrane can originate in MERCS, suggest that the alterations in autophagy observed in AD may be connected with changes in MERCS. Curiously, the MERCS proteins TOM70, Mfn1 and Mfn2 have been shown to be downregulated in SAD [114,209] and FAD [84]. In fact, Mfn2 seems to have a vital role in neurons. First, a mutation in this protein causes Charcot-Marie-Tooth Disease Type 2A (CMT2A) [210]. In addition, conditional knockout of Mfn2 in the adult mouse forebrain led to alterations in mitochondrial dynamics and distribution and to an increased apoptosis rate of in the hippocampus and neurons [211]. Interestingly, a similar phenotype was observed in different AD models [147,212].

**Figure 1:**
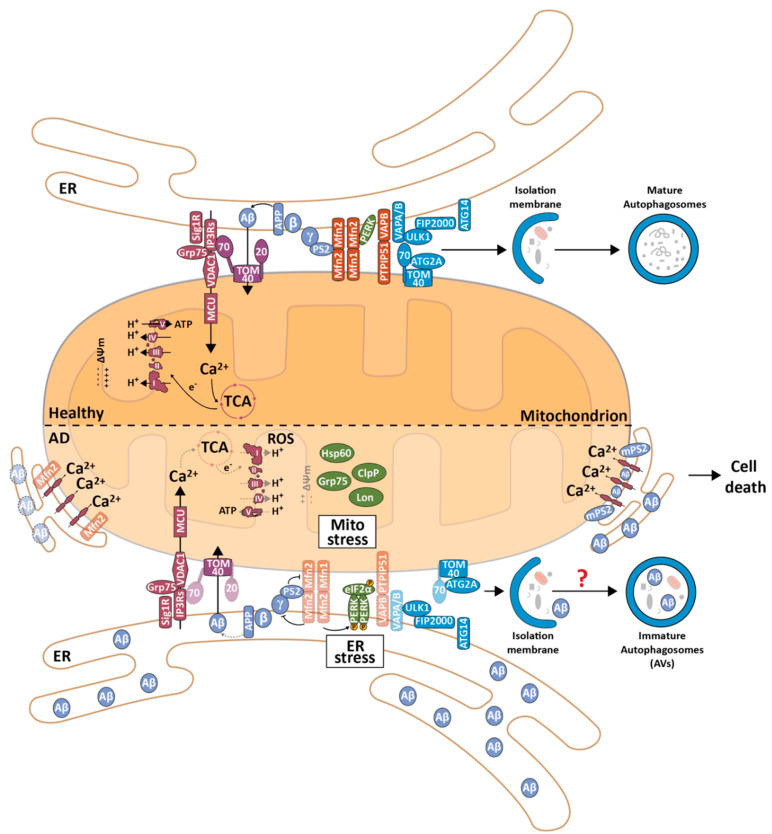
MERCS in health and in AD. In healthy controls (upper part of the figure), different functions of MERCS are integrated to maintain cell homeostasis. The IP3Rs-Grp75-VDAC1 complex together with the MCU complex allows the entry of Ca^2+^ into mitochondria, where it can boost the TCA cycle, inducing ATP production. This complex, together with Mfns and the PTPIP51 and VAPB pair, can modulate the connectivity between the ER and mitochondria. Additionally, the formation of the isolation membrane, which is the precursor to the mature autophagosome, originates at MERCS and can be modulated by the function or ultrastructure of MERCS. Similarly, Aβ also originates in this subcellular region. In AD, the connectivity between the ER and mitochondria is enhanced and Ca^2+^ inside mitochondria is upregulated, and mitochondrial dysfunction, activation of the UPR^ER^ and UPR^mt^, impaired autophagosome maturation and changes in Aβ levels are increased, which can ultimately lead to cell death. Different colours correspond to proteins involved in different cellular processes and faded colours as well as dashed lines represent the downregulation of the process or protein level/function. mPS2 represents PS2 with a FAD mutation.

An illustration of the alterations in MERCS associated with different AD models is provided in Figure 1. In summary, most of the published data suggest that upregulation of MERCS is a hallmark of both SAD and FAD. However, some data indicate the opposite conclusion. Moreover, alterations in Aβ influence the ultrastructure and function of MERCS, but alterations in MERCS affect Aβ levels. However, whether this increase in MERCS is a cause or a consequence of an increase in Aβ and whether it is critical for the neurodegeneration observed in AD remain to be shown.

## 4. MERCS in Parkinson’s Disease

PD is the second most common ND and is associated with tremors, rigidity, bradykinesia and, in the more severe stages of the disease, cognitive impairment. PD is characterized by the loss of dopaminergic neurons in the substantia nigra pars compacta in the midbrain, and similar to AD, misfolded proteins accumulate, including α-synuclein, which forms intracellular Lewy bodies [213].

α-Synuclein has been shown to localize to MERCS, and its overexpression leads to an increase in MERCS [214]. However, PD-associated A53T and A30P mutations lead to a decrease in α-synuclein in MERCS, decreasing the connectivity between the ER and mitochondria, as well as increasing mitochondrial fragmentation [215]. Although the majority of PD cases are idiopathic, the aforementioned mutations in α-synuclein have been shown to cause a familial form of PD. Similarly, mutations in leucine-rich repeat kinase 2 (LRRK2), protein deglycase DJ-1, PINK1 and Parkin also cause familial forms of PD [216]. As mentioned before, PINK1 and Parkin are modulators of mitophagy. PD mutations in these proteins have been reported as loss-of-function mutations that impair the normal functioning of mitophagy and therefore prevent the clearance of damaged mitochondria. Hence, it is not surprising that normal mitochondrial functioning is impaired in PD [217].

LRRK2 has been shown to modulate PERK activity, which then modulates the ultrastructure of MERCS and IP3R-VDAC1-dependent Ca^2+^ shuttle from the ER to mitochondria by phosphorylating and activating Parkin. This leads to the ubiquitination of the MERCS-protein Mfn2 and inducing its proteasomal degradation [218]. Interestingly, DJ-1, PINK1 and Parkin have also been shown to be present at MERCS and to modulate contact. DJ-1 was recently shown to interact with IP3R3-Grp75-VDAC1 and indirectly affect MERCS. DJ-1 PD-associated mutations leads to its loss of function and therefore a decrease in the connectivity between the ER and mitochondria. Ablation of DJ-1 led to impaired IP3R3-Grp75-VDAC1 complex formation and accumulation of IP3R3 in MAMs, while the levels of Sigma-1R were decreased. [219]. Accordingly, overexpression of DJ-1 increases MERCS and Ca^2+^ shuttling from the ER to mitochondria. Moreover, concomitant overexpression of DJ-1 and Mfn2 rescues p53-induced mitochondrial dysfunction and fragmentation [220]. PINK1 also localizes at MERCS in human cells upon mitochondrial uncoupling and the induction of mitophagy [221]. In general, PD mutations in any of the aforementioned proteins impair the normal functioning of mitophagy (a more comprehensive review on this topic was recently published by Liu and colleagues [222]). In MEFs, Parkin indirectly modulates the ultrastructure of MERCS and Ca^2+^ shuttling from the ER to mitochondria via ubiquitination of Mfn2, since this posttranslational modification is required for the normal Mfn2 function as a regulator of MERCS [223,224]. Parkin is also known ubiquitylate other MERCS proteins, including VDAC [121]. In *D. melanogaster*, the overexpression of familial PD-mutated Parkin or PINK1 has been shown to activate the PERK branch of the UPR^ER^ and to induce an increase in connectivity between the ER and mitochondria in a mitofusin-dependent manner [105], as well as in a Miro- and mitochondrial Ca^2+^ shuttling-dependent manner [75].

In 2012, postmortem brains obtained from PD patients showed an increase in the UPR^mt^ marker Hsp-60 as well as unfolded mitochondrial respiratory complexes. In addition, *D. melanogaster* overexpressing PD-related mutant PINK1 or Parkin showed similar results, as did a UPR^mt^ model with truncated ornithine transcarboxylase (ΔOTC). ΔOTC leads to the accumulation and aggregation of ornithine transcarboxylase in the mitochondrial matrix, activating UPR^mt^ [225]. Interestingly, the three models are phenocopies of each other, exhibiting mitochondrial aggregation and fragmentation of cristae and decreased climbing ability, survival and mitochondrial function. In addition, ΔOTC activated autophagy in an AMPK-dependent pathway, and the coexpression of WT Parkin with ΔOTC recovered the dysfunctional phenotypes observed in the ΔOTC flies [226]. ΔOTC has also been shown to induce the accumulation of PINK1, recruiting Parkin and inducing mitophagy, and this accumulation and recruitment can be mitigated by Lon protease [227]. In addition, expression of ΔOTC under the tyrosine hydroxylase (Th) promoter in mice causes neurodegeneration in dopaminergic neuron with dysfunctional motor behaviour. Knocking out PINK leads to a worsened phenotype [228]. Furthermore, loss of Grp75 leads to activation of UPR^mt^ via the upregulation of Hsp-60 as well as activation of mitophagy and apoptosis. Concomitant loss of Grp75 in ΔOTC-overexpressing SH-SY5Y cells exacerbated these phenotypes, while overexpression of either PINK1 or Parkin attenuated them [229]. These data suggest that the mitochondrial dysfunction caused by the accumulation of ΔOTC is similar to that observed in the PD models; therefore, one might assume that modulation of MERCS can also have an effect in ameliorating this phenotype. 

**Figure 2:**
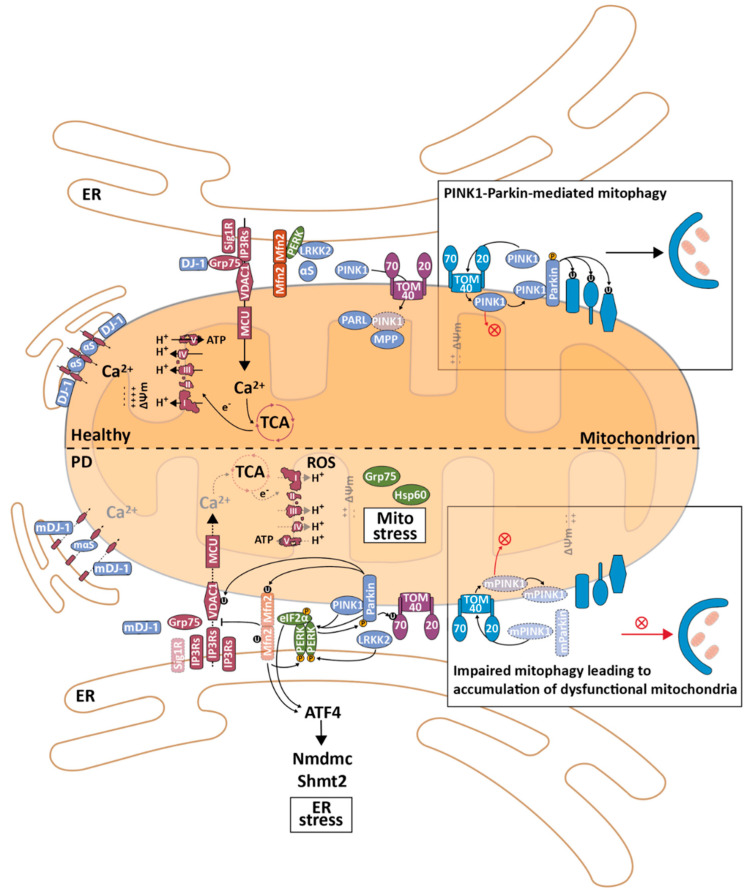
MERCS in health and in PD. In healthy controls (upper part of the figure), different functions of MERCS are integrated to maintain cell homeostasis. The IP3Rs-Grp75-VDAC1 complex together with the MCU complex allows the entry of Ca^2+^ into mitochondria, where it can boost the TCA cycle, inducing ATP production. This complex, together with Mfn2, can also modulate the connectivity between the ER and mitochondria. Under normal conditions, PINK1 is imported into mitochondria, where it can be degraded by PARL and MPP. In cases where mitochondria are damaged, PINK1 import is unsuccessful, and this protein accumulates in the OMM, where it recruits Parkin. Parkin then ubiquitinates proteins at the OMM, inducing mitophagy. In familial PD, mutation in α-synuclein (αS in the figure) increases MERCS, inducing Ca^2+^ overflow inside mitochondria. However, PD-related mutations in DJ-1 (mDJ-1), α-synuclein (mαS), PINK1, Parkin and LRKK2 led to a decrease in MERCS. Nevertheless, these mutations lead to mitochondrial dysfunction, including mitochondrial stress and decreased ATP production as well as ER stress. PD mutations in PINK1 and Parkin (mPINK1 and mParkin in the figure) are usually associated with loss of function and therefore lead to impaired mitophagy activation, preventing the clearance of damaged mitochondria. Different colours correspond to proteins involved in different cellular processes and faded colours as well as dashed lines represent the downregulation of the process or protein level/function. Red arrows as well as red cross correspond to blocked/impaired process.

An illustration of the alterations observed in MERCS associated with PD is provided in Figure 2. However, data in the field are still not coherent, since some publications report that overexpression of WT Parkin leads to an increase in MERCS [223], while others report that the overexpression of loss-of-function mutant Parkin and PINK1 leads to the same increase in connectivity between the ER and mitochondria [105,230], or that knocking down PINK1 leads to reduced MERCS [231]. Regardless, it is quite evident that α-synuclein, LRRK2, DJ-1, PINK1 and Parkin can affect the connectivity between the ER and mitochondria. In addition, the effects of DJ-1, PINK1 and Parkin on MERCS are Mfn2-dependent, enhancing the importance of this protein not only in AD, but also in PD. However, one should keep in mind that the majority of the aforementioned studies used PD models based on a single PD mutation when, in fact, familial PD accounts for only a very small percentage of total PD patients. Further studies need to be performed to elucidate the roles of MERCS in idiopathic PD.

## 5. MERCS in Amyotrophic Lateral Sclerosis and Frontotemporal Dementia

Although ALS is a motor-neuron disease and FTD is a form of dementia, both have been clinically, genetically and pathologically linked. Deposits of fused sarcoma (FUS) and Tar DNA-binding protein 43 (TDP-43) have been reported to be hallmarks of both of these pathologies. Furthermore, mutations in any of these proteins have been connected with the familiar form of ALS/FTD [232]. Both overexpression of TDP-43 and FUS have been shown to disrupt VAPB-PTPIP51 tethering and the ultrastructure of MERCS, as well as the movement from Ca^2+^ from the ER to mitochondria due to activation of glycogen synthase kinase-3β (GSK-3β) [233,234,235]. However, mutation in VAPB P56S, which is known to cause familial ALS type-8, has been shown to accumulate and to increase MERCS [44]. Additionally, in ALS/FTD, both increases and decreases in MERCS have been reported, similar to AD and PD.

As mentioned above, Sigma-1R is a well-known protein in MERCS that acts as a chaperone for IP3R [63], and a mutation in Sigma-1R has been connected with juvenile ALS [236]. More recently, a new mutation in Sigma-1R was shown to induce a new form of juvenile ALS. This mutation led to the accumulation of Sigma-1R in MERCS and to the mislocalization of IP3R, preventing the binding of these two proteins, resulting in deregulated Ca^2+^ homeostasis and decreased ATP synthesis [237]. In mouse models, Sigma-1R knockout led to a decrease in MERCS and induced motor-neuron degeneration, leading to locomotor deficits [238]. Additionally, the knockdown of this protein in primary mouse hippocampal cultures led to neurodegeneration [180].

Interestingly, GSK-3β has also been shown to induce tau phosphorylation and induce tangle-like aggregates similar to those isolated from AD patients [239], suggesting that alterations in the VAPB and PTPIP51 pair may also be involved in AD. In fact, two recent studies showed that these proteins are significantly decreased in SAD [189] and in FAD (the differences were nonsignificant but revealed a tendency) [84]. Additionally, Sigma-1R has been shown to be downregulated in SAD patients [180]. GSK-3β has also been shown to modulate the levels of α-synuclein [240].

**Figure 3:**
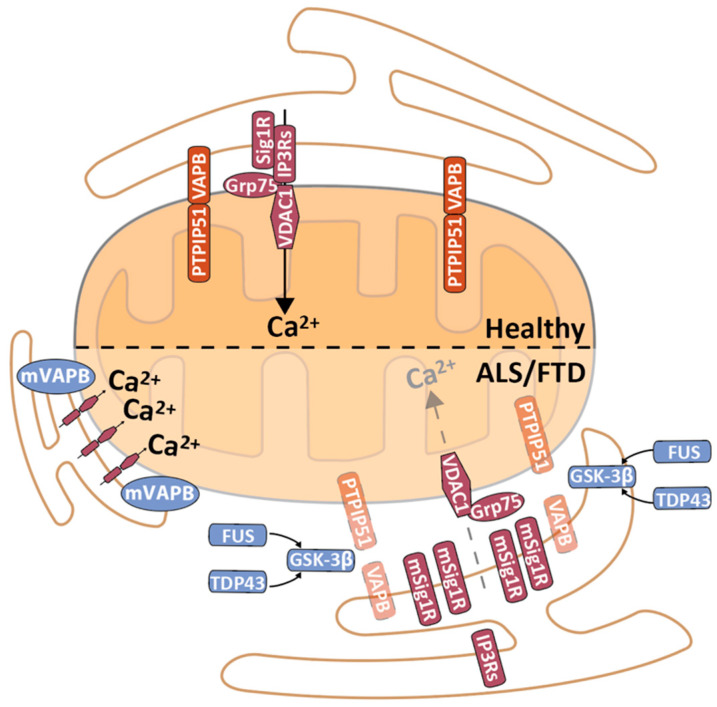
MERCS in health and in ALS/FTD. In healthy controls (upper part of the figure), different functions of MERCS are integrated to maintain cell homeostasis. Complex IP3Rs-Grp75-VDAC1 and VAPB-PTPIP51 modulate the ultrastructure of MERCS. In ALS/FTD, FUS and TDP43 activate GSK-3β, which dissociates PTPIP51 and VAPB and decreases the connectivity between the ER and mitochondria. Mutation in Sigma-1R (mSig1R) leads to the accumulation of these proteins at MERCS and to mislocalization of IP3R outside MERCS, leading to a juvenile form of ALS. Mutation in VAPB (mVAPB) leads to an increase in MERCS and an increase in the flow of Ca^2+^ inside mitochondria. Different colours correspond to proteins involved in different cellular processes and faded colours as well as dashed lines represent the downregulation of the process or protein level/function.

An illustration of the alterations observed in MERCS associated with FTD/ALS is provided in Figure 3.

## 6. MERCS as a Drug Target

Taken together, the data presented above suggest that even though AD, PD, ALS and FTD are four distinctive diseases, alterations of MERCS are common features observed in all of them. Therefore, one can question whether chemical modulation of these MERCS can be used to prevent neurodegeneration and halt the progression of these pathologies. The value of MERCS as drug targets becomes more evident because several clinical monotherapy trials for these pathologies have failed. Therefore, researchers are starting to change their strategies and drop the “one target, one treatment” approach and try to tackle different aspects altered in these pathologies. However, due to the complexity, organization and dynamics of MERCS, finding a drug that allows their modulation and, particularly, a single function/type of contacts might be extremely challenging. Thus, structural system pharmacology, which combines large-scale experimental studies with computational modelling, has been suggested as a possible method to develop efficient drugs to modulate MERCS [37]. In agreement with Magalhães Rebelo and colleagues, we believed that drugs can affect MERCS mainly via three different pathways: by direct interaction with proteins in MERCS, by affecting protein expression levels or by modulating upstream signalling pathways that result in the alteration of the ultrastructure and/or function of MERCS [37]. Currently, there are several drugs available that affect proteins associated with MERCS. However, in most cases, MERCS were not assessed when these drugs were developed, and most of these drugs were discovered in cancer settings. Some examples are as follows:

VDAC1—Drugs that affect the channel conductance (e.g., König’s polyanion, dicyclohexylcarbodiimide, fluoxetine, aspirin and itraconazole), its interaction with its partner hexokinase (e.g., 3-bromopyruvate and methyl jasmonate) or adenine nucleotide translocase (ANT) (e.g., lonidamine, arsenites and steroid analogues) or that affect its own level (e.g., endostatin, myostatin, hierridin B) have been described by Magrì and colleagues [241].

IP3R—As discussed above, Xestospongins B and C are known to block IP3R, affecting several functions of MERCS, such as autophagy and Ca^2+^ homeostasis. Additionally, 2-aminoethyldiphenyl borate has been shown to block IP3R in neurons and increase neuronal excitability [242]. Similarly, trifluoperazine, an FDA-approved antipsychotic drug for schizophrenia, has been shown to induce Ca^2+^ release from IP3R1 and IP3R2 in glioblastoma cell lines [243].

Mfn2—Small molecules and mini-peptides have been developed to alter Mfn2 conformation and its interaction with other proteins, improving mitochondrial defects in the CMT2A model [244,245]. Resveratrol has been shown to improve mitochondrial fitness and to decrease Aβ levels in the CSF of AD patients [246], to increase the levels of Mfn2 [247], to induce Ca^2+^ shuttling from the ER to mitochondria and to enhance the MERCS in cancer cells [248]. However, no study has connected the changes in Mfn2 levels with alterations in the ultrastructure of MERCS upon resveratrol treatment of neuronal cells. In contrast, nicotine has been shown to decrease the levels of Mfn2 [249], decrease OXPHOS and reduce the levels of superoxide anion [250,251]. While resveratrol can boost mitochondrial OXPHOS, as observed in an AD context, the reduction in ROS observed with nicotine treatment may also be helpful. Therefore, treatment with either of these drugs can help ameliorate the phenotypes observed in ND, and further studies must be performed to better understand how modulation of Mfn2 can be applied to therapeutics.

PTPIP51—LDC-3/dynarrestin was first identified as an inhibitor of cytosolic dynein 1 and 2, which blocks endosome movement and affects mitosis in vivo by disturbing spindle orientation. In a new study, this drug was shown to enhance the phosphorylation of PTPIP51, increase the PTPIP51-VAPB interaction and lead to decreased cell viability (as measured by MTT assay) [252,253]. However, how these alterations affect MERCS function and the effect of this drug in ALS/FTD models have not been explored.

Notably, several of the aforementioned drugs were developed in the context of therapies for cancer. While cancer cells tend to avoid cell death and show a general increase in metabolism, in ND, the opposite is usually observed. For example, it was recently shown that the FAD mutation PS2-N141I leads to a decrease in mitochondrial Ca^2+^ and a decrease in the dissociation of hexokinase-1 in mitochondria, leading to a decrease in mitochondrial function [254]. In this case, adding 3-bromopyruvate and methyl jasmonate would cause greater dissociation of the interaction between hexokinase and VDAC1, probably worsening cell fitness. In addition, some of these drugs are known to affect proteins related to MERCS, but in the majority of the studies mentioned, the true ultrastructure and function of MERCS were not measured. However, examples such as itraconazole have been shown to block VDAC1, affecting mitochondrial function and ATP production and inducing autophagosome formation [255], strongly suggesting that the ultrastructure and other functions of MERCS are also altered.

## 7. Conclusions and Implications

In this review, we aimed to summarize the role of MERCS in some of the most common NDs, particularly in AD. During the past 10 years, several findings have been reported regarding how MERCS is altered and affected in these pathologies. In AD, most of the published reports point out that the connectivity between the ER and mitochondria is upregulated, leading to alterations in functions related to MERCS, including Ca^2+^ shuttling from the ER to mitochondria, autophagosome formation and Aβ formation. In addition, models with high levels of Aβ show increased MERCS. However, there are also studies showing a decrease in ER and mitochondria connectivity. It is important to remember that the total connectivity between the ER and mitochondria is based on the sum of all MERCS. If we assume that there are different types of MERCS, as Giacomello and Pellegrini suggested [23], one can assume that when certain types of MERCS are upregulated, others may be downregulated. This possibility may also explain the apparent contradictory data found in the literature that depend on the types of contacts and how the contacts were assessed in a particular study. Nevertheless, whether the alterations of MERCS are a cause or a consequence of cell dyshomeostasis observed in ND remains to be determined.

**Figure 4:**
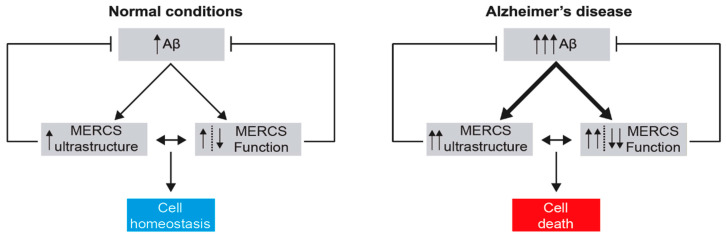
Effect of Aβ in MERCS under normal and AD conditions. Schematic representation of what happens in the cell upon an increase in Aβ levels in normal and AD conditions. Under normal conditions, an increase in Aβ leads to an increase in the connectivity between the ER and mitochondria, affecting MERCS ultrastructure and function. Together, the increased connectivity and altered function of MERCS prevent further Aβ formation. However, when the levels of Aβ are too high, this negative feedback loop is not enough to decrease the levels of this peptide. Aβ continues to induce an increase in the connectivity between the ER and mitochondria, which culminates in Ca^2+^ overflow into mitochondria, activating cell death. Arrows point up correspond to up-regulated and arrows point down down-regulation. Two arrows side-by-side correspond to a further increase or decrease biological process as compare to normal conditions.

In summary, considering the studies on AD and MERCS mentioned in this review, we propose the following updated model of the hypothesis of the function of MERCS in AD: Aβ itself upregulates the connectivity between the ER and mitochondria, and this increase in MERCS halts Aβ production by impairing γ-secretase assembly and activity in a negative feedback loop. However, when an increase in MERCS is sustained due to high levels of Aβ, the normal functions of MERCS are further enhanced, culminating in organellar stress and overflow of Ca^2+^ into mitochondria, leading to organelle failure and cell death (Figure 4).

We would also like to point out that most of the studies presented in this review use either nonneuronal, immortalized or even cancer cell lines. These kinds of cells present a different mitochondrial metabolic wiring than neurons since they undergo the Warburg effect [256,257]. Therefore, it is extremely important that new studies that investigate the role of MERCS in ND are performed in neuronal cells. In fact, some recent studies have used neuronal cells, but very little is known about the effect of MERCS, for example, in microglial cells. A new study showed that immortalized (possibly because they underwent the Warburg effect) astrocytes derived from an AD mouse model showed increased mitochondrial function, increased ROS, decreased mitochondrial Ca^2+^, increased short distance MERCS (8–10) and UPR^ER^ activation. However, interestingly, the authors could not explain the discrepancy between the increase in MERCS and the decrease in mitochondrial Ca^2+^ and function [258].

Although many proteins related to MERCS have been identified, we still do not fully know the complete MERCS proteome or fully understand how these proteins are regulated. One of the major reasons for this lack of understanding is methodological limitation. For example, the methods that allow us to study the dynamics of these contacts lack spatial resolution, and the methods that offer spatial resolution do not allow us to study dynamics since they usually require fixation of the sample. Current methods used to study MERCS have been recently described in several review articles, including those of Scorrano et al. and Giamogante et al. [11,259]. Therefore, further development of new tools to study MERCS will help us to better answer questions such as those related to the possibility of different types of MERCS and whether these MERCS have different proteomes and phospholipid/lipid compositions in the same cell type or in different tissues. Other relevant questions in the field include: Are different types of MERCS affected in different diseases, and are MERCS altered the same way in the early and late stages of a pathology? Further studies in the future might be able to answer these questions.

## Data Availability

Not applicable.
